# Oscillatory mechanics at birth for identifying infants requiring surfactant: a prospective, observational trial

**DOI:** 10.1186/s12931-021-01906-6

**Published:** 2021-12-20

**Authors:** Anna Lavizzari, Chiara Veneroni, Francesco Beretta, Valeria Ottaviani, Claudia Fumagalli, Marta Tossici, Mariarosa Colnaghi, Fabio Mosca, Raffaele L. Dellacà

**Affiliations:** 1 NICU, Fondazione IRCCS Cà Granda Ospedale Maggiore Policlinico, Neonatal Intensive Care Unit, Via Commenda 12, 20135 Milan, Italy; 2 Dipartimento di Elettronica, Informazione e Bioingegneria-DEIB Laboratorio di Tecnologie Biomediche-TechRes Lab, Politecnico di Milano University, Milan, Italy; 3 Department of Clinical Sciences and Community Health, University of Milan, Milan, Italy

**Keywords:** Pulmonary surfactant, Neonatal respiratory distress syndrome, Nasal continuous positive airway pressure, Breathing mechanics, Premature infants

## Abstract

**Background:**

Current criteria for surfactant administration assume that hypoxia is a direct marker of lung-volume de-recruitment. We first introduced an early, non-invasive assessment of lung mechanics by the Forced Oscillation Technique (FOT) and evaluated its role in predicting the need for surfactant therapy.

**Objectives:**

To evaluate whether lung reactance (Xrs) assessment by FOT within 2 h of birth identifies infants who would need surfactant within 24 h; to eventually determine Xrs performance and a cut-off value for early detection of infants requiring surfactant.

**Methods:**

We conducted a prospective, observational, non-randomized study in our tertiary NICU in Milan. Eligible infants were born between 27^+0^ and 34^+6^ weeks’ gestation, presenting respiratory distress after birth. Exclusion criteria: endotracheal intubation at birth, major malformations participation in other interventional trials, parental consent denied. We assessed Xrs during nasal CPAP at 5 cmH_2_O at 10 Hz within 2 h of life, recording flow and pressure tracing through a Fabian Ventilator for off-line analysis. Clinicians were blinded to FOT results.

**Results:**

We enrolled 61 infants, with a median [IQR] gestational age of 31.9 [30.3; 32.9] weeks and birth weight 1490 [1230; 1816] g; 2 infants were excluded from the analysis for set-up malfunctioning. 14/59 infants received surfactant within 24 h. Xrs predicted surfactant need with a cut-off − 33.4 cmH_2_O*s/L and AUC-ROC = 0.86 (0.76–0.96), with sensitivity 0.85 and specificity 0.83. An Xrs cut-off value of − 23.3 cmH_2_O*s/L identified infants needing surfactant or respiratory support > 28 days with AUC-ROC = 0.89 (0.81–0.97), sensitivity 0.86 and specificity 0.77. Interestingly, 12 infants with Xrs < − 23.3 cmH_2_O*s/L (i.e. de-recruited lungs) did not receive surfactant and subsequently required prolonged respiratory support.

**Conclusion:**

Xrs assessed within 2 h of life predicts surfactant need and respiratory support duration in preterm infants. The possible role of Xrs in improving the individualization of respiratory management in preterm infants deserves further investigation.

## Introduction

Despite significant advances in neonatal care, the Respiratory Distress Syndrome (RDS) still represents one of the most challenging complications after preterm birth [1]. Poor lung compliance primarily characterizes RDS and provokes alveolar collapse and lung volume de-recruitment, with impaired gas exchange and increased need for pressure support [2, 3]. Both atelectasis and high-volume ventilation lead to inflammation and ventilation-induced lung injury (VILI) [4–6]. Therefore, preterm infants with RDS often require a prolonged course of respiratory assistance and oxygen supplementation, which are both “life-saving” treatments and risk factors for the development of long-term respiratory sequelae [7–9].

Exogenous pulmonary surfactant promotes lung volume recruitment by reducing the surface tension in the peripheral lung [3, 10], and mitigates lung tissues inflammation [11, 12]. Also, the increasing popularity of “lung-protective strategies” such as surfactant administration by INSURE or Less Invasive Surfactant Administration (LISA)/Minimally Invasive Surfactant Administration (MIST) have limited the use of invasive mechanical ventilation [13, 14]. Therefore, exogenous surfactant may enhance the success of non-invasive respiratory support [15]. However, to maximise benefits, surfactant administration should occur as soon as possible after birth [16].

Although the reduction of peripheral lung aeration is the most relevant functional impairment of RDS, current criteria for surfactant administration are based on the fraction of inspired oxygen (F_I_O_2_) required to maintain peripheral oxygen saturation (SpO_2_) within a target range in infants with worsening respiratory conditions [13, 14]. An oxygen demand-based approach is simple and straightforward for applicability in clinical practice, but it assumes that peripheral oxygenation is a direct marker of lung volume recruitment. On the other hand, multiple factors such as cardiovascular transition, impaired haemodynamic and metabolic status, thermoregulation, and infections may affect oxygen demand during the first few hours after birth [17]. Infants with a surfactant-deficient, de-recruited lung have a variable capacity to compensate for hypoxia by increasing transpulmonary pressure with increased work of breathing (WOB) [18], and a mere, qualitative assessment of respiratory distress may delay surfactant administration. Hence, quantitative criteria encompassing a direct assessment of lung volume recruitment status might allow an early, sensitive and specific identification of infants with surfactant-deficient lungs.

In recent years, the Forced Oscillation Technique (FOT)—or “oscillometry”—emerged as a non-invasive tool for evaluating the respiratory system’s mechanical properties [19–23]. In particular, studies demonstrated that the total respiratory system reactance (Xrs) measured at frequencies lower than 10 Hz at a given distending pressure is a sensitive and specific indicator of lung volume recruitment [19, 24]. FOT measurements are suitable at the bedside in preterm infants during invasive [25–29] and non-invasive respiratory support [30], and even during respiratory stabilization in the delivery room [31]. Moreover, oscillometry assessments in the first few days of life can predict respiratory support duration [27, 30].

We hypothesized that the detection of lung volume de-recruitment by FOT might early identify patients requiring exogenous pulmonary surfactant. Therefore, we tested the correlation between early lung function and a high FIO2 requirement in the first 24 h of life. According to current criteria, a good correlation would suggest that infants with poor lung mechanics at 2 h of life are likely to match oxygenation-based criteria for surfactant administration in the following hours. Consequently, the assessment of lung mechanics would allow an earlier administration of surfactant to the same infants that would receive it according to the current method. On the other hand, different indications between the two methods may highlight different sensitivity in identifying surfactant-deficient subjects. Even if the current approach based on oxygen requirement has limitations, to propose new, not yet validated criteria for surfactant administration that differs from guidelines, we first designed a pilot, observational study to compare the novel approach based on lung mechanics with the gold standard. This mandatory first step would allow identifying agreements and differences between the methods and provide essential data for designing large randomized controlled trials to test the clinical impact of the new approach on clinical outcomes.

The objectives of the current study were, therefore: (1) to evaluate whether an early non-invasive assessment of Xrs performed within 2 h of birth discriminates patients that would meet the current clinical criteria for surfactant replacement therapy in the first day of life and (2) to determine a cut-off value, sensitivity and specificity of Xrs for early detection of infants requiring pulmonary surfactant.

## Methods

We conducted a prospective, non-randomized, observational pilot study in our tertiary NICU in Milan. Our ethical committee approved the research protocol in April 2016 (no. 462_2015). We recruited the study participants between December 2016 and February 2020, whose follow-up lasted until discharge home.

Inborn infants between 27^+0^ and 34^+6^ weeks’ gestation were eligible after obtaining parental consent. Ineligibility criteria included: major congenital abnormalities, intubation before the study entry, neonatal ARDS [32] and participation in interventional studies. Participant selection followed a convenience series based on the timely availability of the research team.

Participants’ clinical management followed institutional guidelines. Infants with signs of respiratory distress had a chest radiograph or lung ultrasounds and received nasal CPAP (NCPAP) for Silverman score > 5 or F_I_O_2_ > 0.30 to target SpO_2_ 89–93% in infants ≤ 32^+6^ weeks or 90–95% in infants > 32^+6^ weeks. Surfactant administration (Curosurf, Chiesi, Parma, Italy 200 mg/kg) occurred by INSURE technique if F_I_O_2_ > 0.40 to target SpO_2_ [33], or because of a sustained increase in F_I_O_2_ associated with a Silverman score > 6 after NCPAP initiation. Criteria for starting MV were persistent F_I_O_2_ > 0.40 to target SpO_2_ after surfactant therapy; apnea spells > 4 in 1 h or > 2 requiring positive pressure ventilation; pH < 7.20 and pCO_2_ > 70 mmHg after receiving NCPAP; or as deemed by clinicians for deteriorating clinical conditions.

Antenatal and perinatal variables were available from the electronic medical records. We considered the following clinical outcomes: administration of surfactant within 24 h; duration of non-invasive respiratory support and MV; the rate of air-leaks, bronchopulmonary dysplasia [34], prematurity-related complications (air-leak syndromes, respiratory support at 36 weeks of gestation, sepsis, patent ductus arteriosus, intraventricular haemorrhage, necrotizing enterocolitis); and length of hospital stay.

We assessed respiratory mechanics at 2 ± 1 SD h from birth. Pressure oscillations at 10 Hz were delivered through a nasal mask over 5 min of quiet breathing during NCPAP at 5 cmH_2_O (Fabian HFO, Vyaire, USA). The FOT frequency was set at 10 Hz as the newborns breathing frequency is higher compared to children and adults and it may interfere with the 5 Hz FOT signal used in previous studies [27]. Flow and pressure tracing were provided by the mechanical ventilator at 200 Hz and recorded on a laptop for subsequent off-line analysis. Xrs was computed using the least squared method [35]. During the procedure, clinicians adjusted F_I_O_2_ to maintain SpO_2_ targets. As Xrs was computed off-line, the medical team was blinded to FOT results.

We evaluated the prediction accuracy of Xrs for identifying infants who received surfactant within 24 h of life (Surf < 24 h group) from those who did not (No-Surf < 24 h group) using the area under the receiver operating characteristic curve (AUC-ROC). We calculated the cut-off value by the J-index. For the sample size calculation, we considered a type I error of 0.05, power 0.85, AUC 0.75 and hypotesised that 25% of infants will receive surfactant, obtaining an estimated sample size of 55 infants. Allowing a dropout rate of 10%, we estimate a sample size of 61.

We additionally divided infants that did not received surfactant before 24 h according to subsequent respiratory history: “better respiratory outcome” (BRO) were infants that never required respiratory support (spontaneous breathing, SB) or received non-invasive respiratory support < 28 days (“short-NIV”); and “worse respiratory outcome” (WRO) included infants who required surfactant or invasive mechanical ventilation (any time) or non-invasive support > 28 days (“long-NIV or Surf > 24 h”). We calculated the AUC-ROC for characterizing Xrs to identify the BRO and WRO infants.

We expressed continuous variables as median [IQR]. We compared continuous variables among more than 2 and 2 groups by Kruskal–Wallis One Way Analysis of Variance on Ranks test and Mann–Whitney Rank Sum test, respectively. We compared dichotomous variables by Fisher Exact tests and expressed them as number (percentage).

## Results

**Fig. 1:**
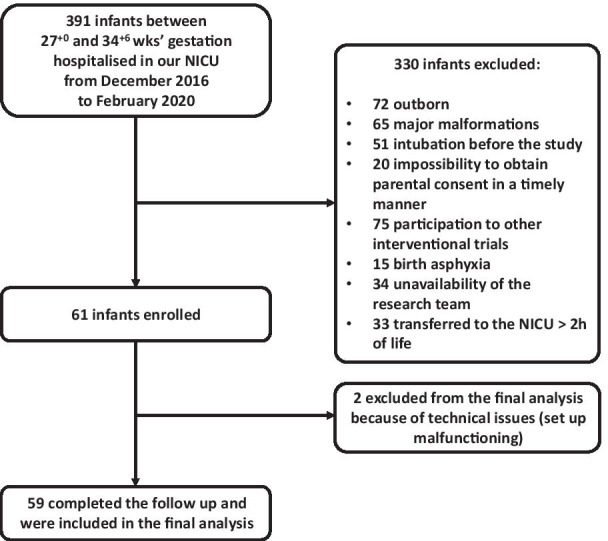
The diagram represents patients’ flow. We reported the overall number of potentially eligible infants (born between27^+0^ and 34^+6^ weeks' gestation, hospitalized in our NICU—which is a reference center for fetal and postnatal surgery—during the study period), the infants excluded (with reasons for exclusion), those confirmed for eligibility and included in the study, completing follow-up, and analyzed. Some infants presented ≥ 1 reason for exclusion

The flow diagram (Fig. 1) represents the eligible infants hospitalized in our NICU during the study period and those excluded with reasons for non-participation. All 61 infants enrolled well-tolerated the study protocol and were able to complete the follow-up. We excluded two subjects from the final analysis for technical issues with the flow and pressure tracing (set-up malfunctioning).

Table 1 reports the two groups' demographic and clinical characteristics (Surf < 24 h and No-Surf < 24 h). Infants who received surfactant within 24 h from birth had a higher but not statistically significant need for resuscitation at birth and a longer duration of non-invasive and invasive respiratory support. The other outcomes explored did not differ significantly. Table 2 shows the participants' variables for the four groups (spontaneous breathing, short-NIV, long-NIVorSurf > 24 h and Surf < 24 h groups).

**Table 1:** Demographic and clinical characteristics of the study population

	Overall population	Surf < 24 h	No-Surf < 24 h
Demographics			
N subjects	61	14	47
Gestational age, median [IQR], weeks	31.9 [30.3; 32.9]	31 [30; 32]	32 [30; 33]
Birth weight, median [IQR], g	1490 [1230; 1816]	1470 [1280; 1780]	1610 [1230; 1947]
Small for gestational age, n (%)	7 (11)	0 (0)	7 (15)
Female, n (%)	32 (52)	10 (71)	22 (47)
Twins, n (%)	44 (72)	9 (64)	35 (74)
Cesarian Section, n (%)	55 (90)	14 (100)	41 (87)
Antenatal steroids, n (%)	59 (97)	14 (100)	45 (96)
Resuscitation at birth, n (%)	51 (83)	14 (100)	37 (79)
Apgar score at 5’, median [IQR]	9 [8; 9]	9 [8; 9]	9 [8; 9]
Arterial cord pH, median [IQR]^a^	7.31 [7.27; 7.35]	7.36 [7.29; 7.36]	7.30 [ 7.27; 7.33]
Clinical characteristics			
Duration of non-invasive RS, median [IQR], days	4.1 [1.5; 29.2]	8.0[5.7; 25.3]	3.0 [1.0; 29.6]*
Duration of mechanical ventilation, median [IQR], days	0.0 [0.0; 0.0]	0.0 [0.0; 0.4]	0.0 [0.0; 0.0]*
Surfactant, n (%)	17 (32)	14 (100)	3 (6)*
Second surfactant dose, n (%)	4 (7)	2 (14)	2 (4)
Air-leak, n (%)	1 (2)	0 (0)	1 (2)
Respiratory support at 36 weeks of gestation, n (%)	10 (16)	2 (14)	8 (17)
Sepsis, n (%)	9 (15)	3 (21)	6 (13)
Patent doctus arteriosus, n (%)	8 (13)	4 (29)	4 (8)
Intraventricular haemorrhage, n (%)	0 (0)	0 (0)	0 (0)
Necrotizing enterocolites, n (%)	5 (8)	2 (14)	3 (6)
Hospitalization, median [IQR], days	34.0 [22.0; 57.7]	36.0 [29.0; 61.0]	33.0 [22.0; 54.0]

The table shows the demographic and clinical characteristics of the overall study population and of the two groups: infants who received surfactant < 24 h of life and infants who did not

^a^Missing data

*p < 0.05 versus Surf group

**Table 2:** Demographic and clinical characteristics for the 4 groups (spontaneous breathing, short-NIV, long-NIV or Surf > 24 h and Surf < 24 h) and for the 2 groups (Better and Worse Clinical Outcome) according to the subsequent clinical outcomes

	Spontaneous breathing	Short-NIV	Long-NIV or Surf >24	Surf <24	BRO	WRO
Demographic						
N subjects	8	23	16	14	31	30
Gestational age, weeks	32.7 [32.1; 33.3]	32.9 [32.1; 33.1]	30.0 [29.2; 30.6]*°	31.1 [30.4; 31.9]*	32.9 [32.1; 33.2]	30.4 [29.6; 31.3]^#^
(min.–max.)	(29.0; 33.6)	(29.0; 34.7)	(27.9; 32.7)	(27.8;34.1)	(29.0;34.7)	(27.8;34.1)
Birth weight, g	1895 [1695;2215]	1720 [1411; 1997]	1220 [900; 1345]*°	1470 [1280; 1780]	1730 [1479; 2007]	1285 [1100; 1490]^#^
	(1090–2495)	(1180–2345)	(745–2375)	(900–1955)	(1090–2495)	(745–2375)
Small for gestational age	0 (0)	3 (13)	4 (25)	0 (0)	3 (10)	4 (13)
Female	3 (37)	9 (39)	10 (62)	10 (71)	12 (39)	20 (67)^#^
Twins	6 (75)	19 (83)	10 (62)	9 (64)	25 (81)	19 (22)
Cesarian Section	6 (75)	19 (83)	16 (100)	14 (100)	25 (81)	30 (100)^#^
Antenatal steroids	7 (87)	22 (96)	16 (100)	14 (100)	29 (93)	30 (100)
Resuscitation at birth	1 (12)	20 (87)°	16 (100)°	14 (100)°	21 (68)	30 (100)^#^
APGAR score at 5’	9 [9;10]	9 [8; 9]	9 [8; 9]	9 [8; 9]	9 [9; 9]	9 [8; 9]
Arterial cord pH	7.31 [7.31; 7.33]	7.36 [7.35; 7.39]	7.37 [7.35; 7.40]	7.40 [7.36; 7.40]	7.30 [7.25; 7.32]	7.32 [7.27; 7.36]
Clinical characteristics						
Duration of non-invasive RS, days	0 [0; 0]	2[1.2; 3.4]	37.6 [29.4; 47.0]*°	8 [5.7; 25.3]*°	1.8 [0.1; 3.2]	29.4 [6.2; 44.0]^#^
	(0-0)	(0.5-7.1)	(1.5-108.3)	(1.5-59.2)	(0-7.1)	(1.5-108.3)
Duration of mechanical ventilation, days	0 [0; 0]	0 [0; 0]	0 [0; 0]	0 [0; 0.4]	0 [0; 0]	0 [0; 0]
	(0-0)	(0-0)	(0-14)	(0-4)	(0-0)	(0-14)
Surfactant	0 (0)	0 (0)	3 (19)	14 (100)*°^§^	0 (0)	17 (57)^#^
Second surfactant dose	0 (0)	0 (0)	1 (6)	2 (14)	0 (0)	3 (10)
Air-leak	0 (0)	0 (0)	1 (6)	0 (0)	0 (0)	1 (3)
Respiratory support at 36 weeks gestation	0 (0)	0 (0)	8 (50)*	2 (14)	0 (0)	10 (33)^#^
Sepsis	0 (0)	1 (4)	5 (31)	3 (21)	1 (3)	8 (27)^#^
Patent doctus arteriosus	0 (0)	1 (4)	3 (19)	4 (29)	1 (3)	7 (23)^#^
intraventricular haemorrhage	0 (0)	0 (0)	0 (0)	0 (0)	0 (0)	0 (0)
Necrotizing Enterocolites	0 (0)	1 (4)	2 (12)	2 (14)	1 (3)	4 (13)
Hospitalization, days	22 [18; 25]	28 [18; 40]	63 [51; 78]*°	36 [29; 61]	22 [18; 37]	55 [34; 71]^#^
	(12–53)	(7–61)	(25–178)	(18–101)	(7–61)	(18–178)

The table shows the population’s demographic and clinical characteristics divided in 4 groups (on the left) and in 2 groups (on the right) according to the subsequent clinical outcomes

*p < 0.05 versus Short-NIV; ° p < 0.05 versus Spontaneous Breathing; ^§^ p < 0.05 versus Long-NIV or Surf > 24 h; ^#^ p < 0.05 versus BRO

**Fig. 2:**
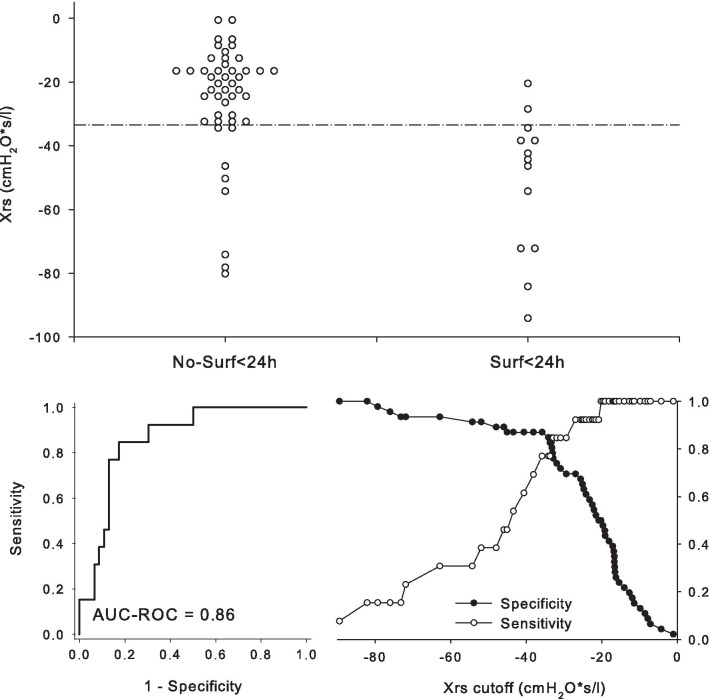
The top panel represents the dot plot of individual respiratory reactance (Xrs) in the two groups: infants receiving surfactant < 24 h from birth (on the right) and those who did not (on the left). The bottom panels represent the area under the receiver operating characteristic curve (AUC-ROC) of Xrs for predicting surfactant administration < 24 h (bottom-left panel) and sensitivity and specificity of Xrs at different values (bottom-right panel). The best-identified cut-off of Xrs was − 33.4 cmH_2_O*s/L

In the dot-plot of Fig. 2, we represented the individual values of Xrs in the Surf < 24 h and No-Surf < 24 h groups. Xrs predicted infants needing surfactant within 24 h from birth with an AUC-ROC of 0.86. The identified cut-off of Xrs was − 33.4 cmH_2_O*s/L, with a sensitivity of 0.85 (0.55–0.98) and specificity of 0.83 (0.69–0.92).

In the No-Surf < 24 h group, eight infants had an Xrs < − 33.4 cmH_2_O*s/L but did not receive surfactant within 24 h. During the subsequent NICU stay, they required longer respiratory support than infants who received surfactant within 24 h from birth (median (IQR) 30 (3; 44) days vs 2 (1; 6) days).

**Fig. 3:**
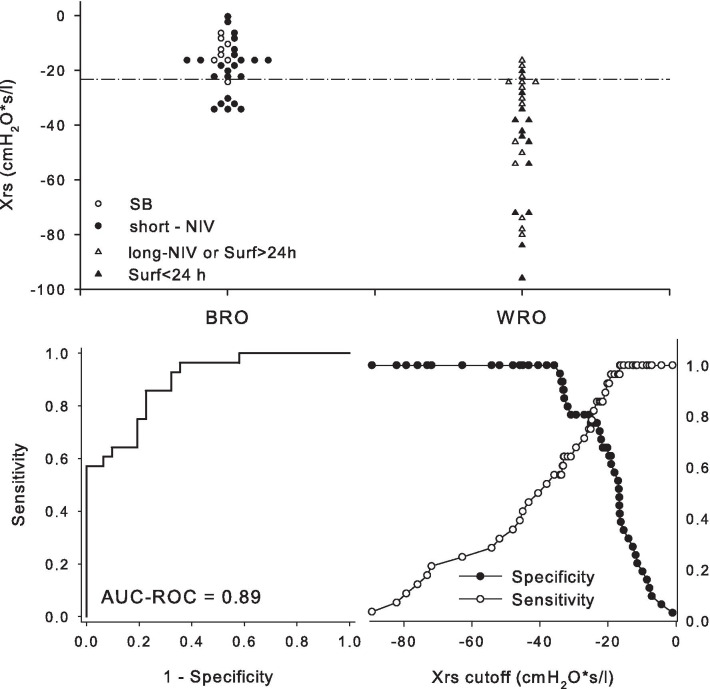
Dot plot of individual respiratory reactance (Xrs) (top panel) for the four groups: spontaneously breathing infants with no support (SB) and infants receiving NIV < 28 days (short-NIV) on the left (better respiratory outcome, BRO); infants receiving surfactant < 24 h (Surf < 24 h) and receiving surfactant after 24 h or NIV > 28 days (NIV-long or Surf > 24 h) on the right (worse respiratory outcome, WRO). The bottom panels show the area under the receiver operating characteristic curve (AUC-ROC) of Xrs for predicting WRO (bottom-left panel) and sensitivity and specificity of Xrs at different values (bottom- right panel). The best-identified cut-off of Xrs was − 23.3 cmH_2_O*s/L

Eight infants (GA: 29.0–33.6) never required respiratory support (SB), 23 (GA: 29.0–34.7) received non-invasive respiratory support < 28 days (short-NIV), 16 (GA: 27.9–32.7) required surfactant after 24 h of life (3 infants) or non-invasive support > 28 days (long-NIV or Surf > 24 h), and 14 (GA: 27.8–34.1) received surfactant within 24 h (Surf < 24 h). In Fig. 3, we illustrated individual Xrs values according to the subsequent respiratory history. The median [IQR] Xrs were − 13 (− 16; 10), − 19(− 27; − 16), − 30(− 52; − 24), − 44(− 71; − 37) cmH_2_O*s/L respectively for SB, short-NIVlong-NIVorSurf > 24 h and Surf < 24 h. Infants who needed surfactant within 24 h and infants who required surfactant after 24 h of life or non-invasive support > 28 days showed lower and more variable Xrs values at 2 h of life. Xrs < − 23.3cmH_2_O*s/L predicted infants with “worse respiratory outcomes” (WRO) with AUC-ROC = 0.89 (Fig. 3), sensitivity of 0.86 (0.67–0.96) and specificity of 0.77 (0.59–0.90).

## Discussion

The main findings of the current study are that in preterm infants receiving NCPAP (1) an “early” lung mechanics assessment by FOT during the postnatal transition is feasible; (2) a Xrs cut-off value of − 33.4 cmH_2_O*s/L detected 85% of infants who received surfactant within 24 h from birth following current oxygenation-based criteria; (3) the same threshold identified 8 patients who did not receive surfactant in the first 24 h of life, despite their poor lung mechanics, because they did not match current criteria for surfactant treatment; interestingly, the vast majority of them developed poor respiratory outcomes, requiring a longer duration of respiratory support as compared to infants with poor lung mechanics who received surfactant; (4) when patients were grouped considering their respiratory outcomes, Xrs at 2 h of life detected patients with ‘worse respiratory outcomes’, (i.e. patients who either received surfactant or required respiratory support for > 28 days) with an AUC-ROC of 0.89. A cut-off value of − 23.3 cmH_2_O*s/L provided a sensitivity of 0.86 and a specificity of 0.77 for identifying worse respiratory outcomes patients.

To the best of our knowledge, this is the first study evaluating the role of early lung mechanics assessment for individualizing surfactant therapy. Most previous studies on lung mechanics in preterm infants focused on correlation with long-term respiratory outcomes, such as the duration of respiratory support or the occurrence of chronic lung disease (CLD) [36–44]. Several approaches have been suggested for assessing lung mechanical properties in preterm infants, including the interrupter technique [39] or the single-occlusion technique [36, 39–42, 44] or by fitting the equation of motion of the respiratory system [37–39, 43]. The need for either the absence of spontaneous breathing or the capability to measure spontaneous respiratory efforts and the complexity of those techniques limit their applicability during the critical phase of preterm infant postnatal adaptation. Pioneering studies showed the feasibility of the forced oscillation technique on preterm infants [24]. Oscillometry is less invasive than other techniques for assessing lung mechanics; it does not require patients' disconnection from the ventilatory circuit nor interfere with the ventilator. The technique does not involve estimating the pleural pressure by positioning an oesophagal balloon or evoking the Hering–Breuer inflation reflex for respiratory muscle relaxation. Previous studies demonstrated FOT feasibility in the delivery room [31] and the NICU, both in intubated patients [27–29] and infants receiving NCPAP [30]. Moreover, thanks to recent technological advancements, oscillometry can be now integrated into modern mechanical ventilators.

Oscillometry performed within 2 h of life identified infants with compromised respiratory mechanics who required the administration [16] of pulmonary surfactant on the first day of life as per standard criteria [33]. Considering that the administration of surfactant occurred at a mean (std) 8 ± 5 h of life, the use of FOT may support a more timely administration, with potential impacts on the efficacy of the therapy [16].

While most infants with poor Xrs matched oxygenation-based clinical criteria for surfactant replacement therapy within the first 24 h of life, several infants did not. These discrepancies may result from the physiological meaning of the two parameters, i.e. lung volume recruitment and oxygenation. Preterm infants with lung volume de-recruitment have a variable ability to compensate for the reduced gas exchange by increasing their WOB. Infants generating high transpulmonary pressure can counterbalance the insufficient lung volume recruitment and limit their oxygen supplementation need in the first few hours after birth. However, developing high transpulmonary pressures may excessively stress the lung tissue and lead to inflammation and patient self-inflicted lung injury [45]. Interestingly, the only patient of our study who developed a pneumothorax (which required chest drain and invasive mechanical ventilation) was one of the infants with poor lung mechanics at birth who did not receive surfactant within 24 h of life because of low oxygen demand. Moreover, increased WOB may result in progressive muscle exhaustion, protracting the need for respiratory support. Since our secondary exploratory analysis on patients’ sub-groups showed that the surfactant group had an overall shorter respiratory support duration than the long-NIV group despite overlapping oscillatory mechanics data, we speculated that some infants of the long-NIV group might have benefited from surfactant therapy, regardless of current indications [13].

Our secondary exploratory analysis also showed that, despite several possible determinants for prolonged respiratory support may develop in the first 28 days of life, if lung reactance is impaired at birth, the risk of negative respiratory outcomes is increased. This is also in line with the finding of other studies which compared FOT data to clinical parameters and respiratory outcomes [27, 30] and found that FOT data in addition to GA improved prediction of days of respiratory support or BPD development. Future investigations should evaluate whether the possibility to identify infants more susceptible to lung injury early at birth can improve tailoring their respiratory management. As FOT can be easily performed, repeating the test with time may also provide useful information on the evolution of the patients in response to different events and medical interventions.

Only 2 out of 13 infants received surfactant without presenting poor Xrs because they matched the current clinical criteria. Surfactant therapy exposes infants to direct laryngoscopy and related side effects (e.g. trauma, bleeding, haemodynamic instability, pain requiring analgesic drugs) even when applied by less invasive methods and imposes high economic and environmental costs (animal-derived product). Therefore, early lung mechanics assessment at birth might allow more appropriate surfactant therapy with economic and ethical benefits.

Metanalysis showed that “prophylactic” surfactant decreases infants mortality, the risk of air leaks, and the composite outcome of BPD or death [46]. Later metanalysis, including studies with more extensive and early use of non-invasive CPAP, demonstrated that “selective” surfactant associates with a lower risk of chronic lung disease or death [47], therefore, “prophylactic” surfactant use had been discontinued. “Early selective” surfactant administration coupled with NCPAP reduces the risk of BPD and air-leak syndromes compared to “later selective” surfactant followed by MV [48]. Moreover, a lower treatment threshold (F_I_O_2_ < 0.45) as compared to a higher one (F_I_O_2_ > 0.45) reduces the rate of air-leak syndromes and BPD [48]. However, to date, no trial directly compared different thresholds of F_I_O_2_ for the first surfactant administration. Kattwinkel et al. [49] compared two thresholds (F_I_O_2_ > 0.30 versus F_I_O_2_ > 0.40) for the second and further doses surfactant. They found no difference in the duration of MV or any long-term respiratory outcomes except for significantly higher mortality in infants with complicated RDS who received treatment according to a high threshold*. Dargaville *et al*.* found that F_I_O_2_ > 0.30 is the critical cut-off, which increases the risk of CPAP failure in infants < 32 weeks gestation with an AUC of 0.80 [50]. Based on these retrospective observations, the authors of the European consensus recently updated the guidelines on surfactant administration suggesting F_I_O_2_ > 0.30 as a threshold for surfactant therapy regardless of GA or other risk factors. However, CPAP failure intended as the need for intubation and endotracheal ventilation is not the only adverse respiratory scenario for preterm infants, as documented by the increasing number of premature infants who develop chronic sequelae receiving minimal or even no MV. Laughon et al*. *[51] identified three respiratory disease patterns among extremely preterm infants based on the F_I_O_2_ required during the first two weeks of life. 20% of infants with low F_I_O_2_ both in the first week and at 14 days have a low incidence of CLD (17%). By contrast, most infants with F_I_O_2_ > 0.25 both during the first and second weeks develop CLD (67%). The remaining infants with low F_I_O_2_ at the start and increased the oxygen requirement by 14 days still have a considerable risk of CLD (51%). These findings demonstrate that oxygen demand alone cannot fully identify the early origins of lung disease in preterm infants.

We believe that hypoxia is only an indirect marker of alveolar recruitment. The capability to compensate for hypoxia may vary among different infants and is not easily predictable by GA alone. Preterm infants with surfactant-deficient lungs may counterbalance alveolar de-recruitment by increasing muscle fatigue, potentially delaying or even denying an effective surfactant administration. By increasing their WOB, these infants also exercise mechanical stress on the lung tissues, generating self-inflicted lung injury [45]. Additionally, relying exclusively on oxygenation for determining surfactant need may lead to useless (and potentially harmful) surfactant treatments. In addition to lung volume recruitment, haemodynamic changes, metabolic status, thermoregulation and inflammation may affect oxygen demand. Limitations of the standard oxygenation-based approach to surfactant therapy have increased the interest for surfactant predictive tests, such as the Lamellar Body Counts on gastric aspirates and the lung ultrasounds, which are still under evaluation [52–54]. Further large multicentre studies should compare Xrs measurements versus the other available techniques for predicting surfactant need [54]. Finally, an earlier and more appropriate surfactant administration may have long-term effects on the preterm lung, protecting the lung parenchyma from excessive mechanical stress and enhancing a more physiological respiratory adaptation long term.

The current study has limitations. First, the participant selection occurred based on the research team's availability due to experience with the experimental set-up. Nonetheless, to limit the observation and confirmation bias, the assessors of lung mechanics were blinded to test results; similarly, the investigators performing the off-line analysis of lung reactance were not aware of patients’ clinical details until the end of the recruitment. Second, the study population's choice of 27–34 weeks' gestation might have excluded infants with more severe RDS. The GA range choice was due to both ongoing competitive trials involving lower GA infants in the Unit and the experimental set-up. Changing the respiratory support device for the study acquisition might have disrupted the more immature infants during the delicate adaptation stage. However, more premature infants receive surfactant in a higher percentage of cases. Whereas in the GA range included in the study, the probability of receiving surfactant is more variable and unpredictable, and the identification of predictors may be more valuable. Finally, we conducted a monocentric study. However, the data acquired in our centre are in line with a previous publication evaluating lung mechanics during NCPAP in the first week of life for feasibility, tolerability and range of measurements (Xrs at 2 days was –26.13 (12.43) cm H2O*s/L). We also confirmed previous research findings, demonstrating a correlation between early lung mechanics assessment and respiratory support duration in intubated [27] and spontaneously breathing infants [30]. Considering the agreement with previous publications, the sample size, and the technique's reproducibility, we believe that our findings may have valid generalizability, even though a comparison within a larger population of similar GA and assistance level is necessary before drawing definite conclusions.

In conclusions, according to our data, a non-invasive assessment of lung mechanics within 2 h of birth is feasible, and Xrs value predicts the need for surfactant as defined by current clinical administration criteria. Xrs also identifies infants who later develop poor respiratory outcomes. Providing an objective evaluation, Xrs combined with oxygenation may help clinicians tailor a more timely and individualized approach to surfactant therapy. Whether the use of early oscillatory mechanics for guiding surfactant therapy in addition to oxygen-based criteria may improve respiratory outcomes of premature infants deserves further evaluation by conducting large multicenter diagnostic accuracy studies.

## Data Availability

The datasets used during the current study are available from the corresponding author on reasonable request.

## Abbreviations

AUC-ROC: Area under the receiver operating characteristic curve
BRO: Better respiratory outcome
BPD: Bronchopulmonary dysplasia
CLD: Chronic lung disease
CPAP: Continuous positive airway pressure
F_I_O_2_: Fraction of inspired oxygen
FOT: Forced oscillation technique
INSURE: Intubation surfactant extubation
IQR: Interquartile range
LISA: Less invasive surfactant administration
MIST: Minimally invasive surfactant administration
MV: Mechanical ventilation
NCPAP: Nasal continuous positive airway pressure
NICU: Neonatal intensive care unit
NIV: Non-invasive ventilation
RDS: Respiratory distress syndrome
SB: Spontaneous breathing
SD: Standard deviation
SpO_2_: Peripheral oxygen saturation
VILI: Ventilation-induced lung injury
WOB: Work of breathing
WRO: Worse respiratory outcome
Xrs: Oscillatory respiratory reactance
